# Listening to Patients With Lupus: Why Not Proactively Integrate the Internet as a Resource to Drive Improved Care?

**DOI:** 10.2196/44660

**Published:** 2023-03-29

**Authors:** Caroline A Blackie, Lisa Gualtieri, Shanthini Kasturi

**Affiliations:** 1 Johnson and Johnson Vision, Inc Jacksonville, FL United States; 2 Department of Public Health and Community Medicine Tufts University Boston, MA United States; 3 Department of Medicine Division of Rheumatology Tufts Medical Center Boston, MA United States

**Keywords:** lupus, patient symptom, patient journey, chronic disease, lived experience, patient experience, patient need, digital health intervention, autoimmune disease, clinical care, digital voice, social media, patient care, online community, social listening, autoimmune, experience, perspective

## Abstract

Systemic lupus erythematosus (SLE) is a multisystem autoimmune disease. It is characterized by a broad spectrum of manifestations, depending on the affected organs and the severity of the inflammation at the time of presentation. Despite improvements in management, treatments are required on a chronic, cyclical basis; have high potential for unpleasant side effects; and deliver variable efficacy. Patients require care from multiple specialists, which can be delivered simultaneously and sporadically. Our fragmented health care system further exacerbates the disconnect between intermittent medical care and the lived experiences of patients with SLE. The goals of this research are to (1) assess the current standard of care for patients with SLE through the review of medical literature, including clinical consensus guidelines and systematic reviews; (2) assess the lived experiences of patients with lupus through the review of peer-reviewed literature on social listening, structured interviews, and data available from the open-access digital health platform PatientsLikeMe; and (3) present the perspective that the medical community has an opportunity to acknowledge and review the use of digital health interventions (DHIs) with their patients. The results of this research indicate that patients are incorporating DHIs, such as the internet and social media platforms, as critical components of their care for even the most basic of support. Although patients with SLE are depending on this support to shape their care, it is not considered a primary source of care by clinicians. Integrating the voices of patients brings valuable dimension to understanding the lived experiences of patients with SLE and the impacts of mutually dependent patient needs as patients navigate the disease in daily life. The medical community has a meaningful opportunity to leverage and recommend existing DHIs, such as web-based community platforms and web-based patient registries, at every stage of the patient journey to help patients better manage their condition. This has the potential to proactively build patient trust and well-being, reduce the underreporting of symptoms, increase shared decision-making, inform and shape clinical guidelines and future research, and improve patient outcomes.

## Introduction

Systemic lupus erythematosus (SLE) is a chronic, multisystem autoimmune disease. It is characterized by a broad spectrum of manifestations, depending on the affected organs (eg, skin, kidney, lung, heart, brain, etc), the chronicity of the disease, and the severity of disease activity at the time of presentation [1]. According to a recent review of several registries in the United States, the reported prevalence of SLE ranges from 43 to 200 people per 100,000 people, depending on ethnicity [2]. A global review of the epidemiology of SLE reveals that both incidence and prevalence depend on ethnicity as well as geography, with a consistent trend toward higher incidence and prevalence in racial and ethnic minority groups; non-Hispanic Black women are the most affected. Additionally, newer data indicate that prevalence rates of SLE are on the rise [3].

## Health Disparity and SLE

The full scope of SLE disease burden has not been fully characterized, but studies show that mortality burden falls disproportionately on racial and ethnic minority groups [4]. This burden is only partially explained by variables such as comorbidities and social determinants of health [3]. When considering the complexity of diagnosis and management for SLE, our fragmented health care system, and the disproportionate lack of access for underrepresented communities [5-7], SLE represents a significant challenge for racial and ethnic minority women.

## Expect the Unexpected

The SLE disease course is predictably unpredictable due to fluctuations in disease flare-up times, the variability in affected organs, and the inability to differentiate between relative inactivity and possible remission [1]. Physicians wrestle with identifying the most efficacious treatments to manage individual clinical manifestations of the disease, and patients must manage both the consequences of the disease and the often-damaging effects of the treatments they are prescribed [8]. Although there is agreement at a high level as to what SLE is, at an individual level, there is limited consensus regarding which precise biomarkers and clinical manifestations can be used to titrate and deliver targeted therapy.

## An Invisible Disease

Chronic diseases, such as SLE, are often invisible. Patients can experience debilitating symptoms but appear healthy [9-12]. For example, patients with SLE may not be able to get out of bed due to crushing fatigue, yet their appearance is unchanged, and there are no existing biomarkers to explain or validate their fatigue. The invisibility of SLE has been highlighted in recent literature due to its predictably negative impact on employment. Patients with SLE have a 4 times increased risk of exiting the workforce [9]. Leveraging a thematic analysis of patient voices, this research also highlights the positive impact of providing a supportive environment for patients with SLE, such as by providing flexible work hours, remote work options, and support during flares and by validating that a patient’s condition is real [10].

## Gaps in Evidence-Based Clinical Consensus Guidelines

Patients require nuanced, individualized, clinical support from multiple different specialties. This is a nontrivial conundrum for both patients and clinicians, which is highlighted by the sparsity of evidence-based clinical consensus guidelines in the literature. A recent update to the European consensus guidelines for SLE [13] and a systematic review of all guidelines for SLE [8] reveal some progress in this area. However, these guidelines call out multiple gaps in evidence for specific manifestations of the disease, along with a variety of unmet patient and physician needs. Attempts to provide generalized guidance are problematic where evidence gaps persist. As an example, the recommendation for the use of a physician global assessment [13] intended to improve long-term outcomes of patients with SLE has been questioned due to high intrarater and interrater variability [14]. Ongoing work to address these gaps does not call for the integration of the patient voice through digital health platforms and interventions. This is likely, in part, because of how the gaps are defined. The primary gaps in clinical consensus guidelines are centered around physicians’ need to better access and prescribe safe and effective therapeutics, and they are not directly focused on the comprehensive lived experiences of patients.

## Treatment

SLE treatments are primarily targeted to the following four key areas: managing flare-ups and periods of high disease activity; preventing flare-ups; ensuring long-term survival (preventing organ damage); and improving patients’ quality of life, which includes managing and minimizing treatment side effects [15]. Advances in genetics and genomics may demonstrate the potential to articulate molecular profiles for SLE and other related conditions, which may facilitate more targeted therapy [16]. Currently, immunosuppressants and corticosteroids, which are frequently associated with negative and lasting adverse effects, remain the core treatments for SLE [17-19].

## Physician Needs

A recent systematic review of current clinical consensus guidelines for SLE outlined several physician needs. These needs were summarized as follows: consensus guidelines for specific types of target tissue inflammation not covered under existing guidelines (eg, serositis, headaches, retinal vasculitis, photosensitivity, and others); social determinants of health as a prognostic tool; more data on ethnicity and its relationship with disease severity; guidance for a lack of medication adherence in treatment; and, the most glaring of all, more patient input when developing guidelines for a disease that is largely diagnosed clinically [8].

## Patient Needs

Due to the complexity of SLE; the variability in patient presentation; and the shortage of evidence-based and well-defined criteria for a definitive diagnosis and targeted, safe, and effective treatment options for SLE, the typical patient journey is a personal, medical, and social quagmire. There have been efforts to capture patient needs via social listening on public forums, such as PatientsLikeMe [20], LupusConnect [21], and others [22-24]. Additionally, in 2017, a large, international, multicenter, observational cohort study was initiated to track patients over a multiyear period, with the intent of monitoring treatment outcomes [25], but also to capture critical patient-reported outcomes, such as quality of life, and track health care utilization [16].

A series of structured interviews conducted with patients with SLE in 2014 validated 7 patient need domains [26]. The physical domain—the most investigated and the most prioritized target for medical intervention—is 1 of 7 domains that were identified as critical for patients with SLE. Clinicians are most attuned to attending to the physical needs domain, which places the burden on patients to describe the full complexity of their lived experiences. The other six domains are the economic, health services, health education, social, emotional, and daily living domains. Such research that uses patients’ experiences as a data source is impactful for informing care. A recent study that tested the feasibility of integrating electronically administered patient-reported outcome measures (PROMs) into the care of patients with SLE demonstrated that patients perceived this approach to be “quite a bit” or “very” useful in communicating their experiences with their clinicians a majority of the time (55% of encounters) [27]. These PROMs covered physical and nonphysical domains, such as anxiety, depression, intimate relationships, and participation in social activities. It is notable that clinicians did not find the integration of the PROMs to be as helpful (17%-20% of encounters) [27].

**Figure 1:**
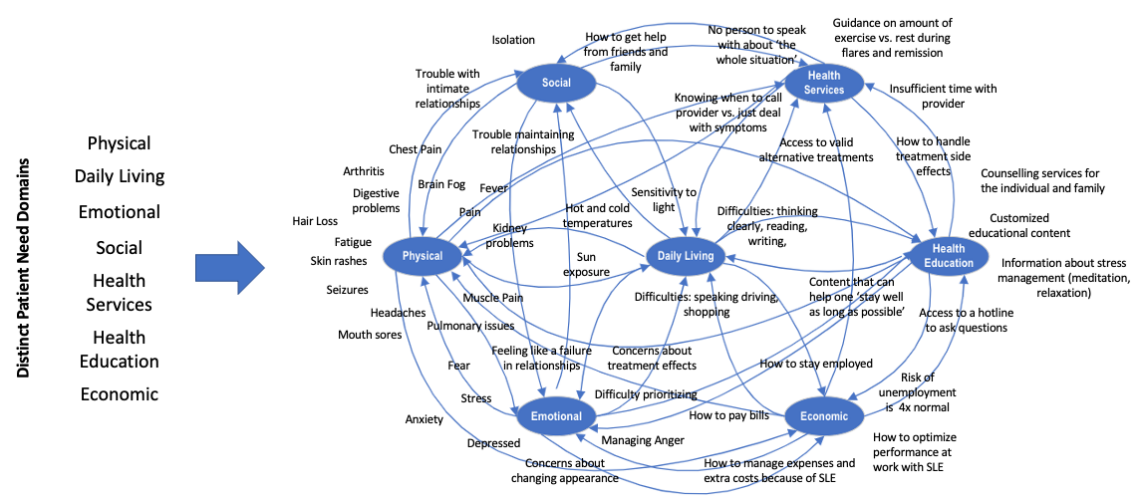
A systems map illustration of the lived patient experience. A multitude of patient digital voices are overlayed over patient need domains. The map is intended to highlight that, from the perspective of patients, their need domains, while distinct, are highly interdependent; any single domain impacts all domains. SLE: systemic lupus erythematosus.

A patient with SLE can experience multiple needs simultaneously, of which all are critical to patient well-being (Multimedia Appendix 1) [28]. Although the needs domains are distinct, an unmet need in any one of these domains will negatively impact each of the other domains. For illustrative purposes, rather than presenting these needs domains in list or tabular form, as is the norm, a systems map approach is shown in Figure 1. Additionally, excerpts of patient needs are superimposed on each of the domains. This approach demonstrates how the failure to address a need in one domain has a systemic impact on the entire patient experience and can affect needs in other domains.

Figure 1 represents an example of how listening to and integrating patient voices help to inform categories of specific patient needs and shows that these needs, while distinct, are not independent. Layering patient voices over these needs domains and illustrating the connections between these domains bring the patient experience to life. For this approach to be fully developed, clinicians need access to tools for hearing and assimilating the integrated lived experiences of patients with SLE.

Another way to demonstrate that physical hardship is only one of multiple struggles among patients with SLE is to capture patient symptoms recorded on a digital platform that is designed for patients to share their experiences. A word cloud of 68,581 symptoms recorded by 341,372 members [20] is represented in Multimedia Appendix 1. The dominant themes in this word cloud are that patients with SLE are depressed, anxious, and fatigued and are in pain.

## Health Care Delivery

The US health care delivery system, broadly speaking, provides intermittent access to care. Intermittent access to care is the backbone of our existing business model of health care. When patients experience a problem, they interface with the health care system, obtain an assessment, and receive a treatment or a recommendation for how to manage their condition. Patients then “exit” the system and test the recommendation, and the cycle repeats as needed. When a disease is well characterized, predictable, and acute, this delivery system works; SLE is none of these. The between-care, individual-level manifestations and daily fluctuations experienced by patients with SLE can impact their lives in a multitude of ways that cannot be easily captured during intermittent and brief interactions with clinicians. Additionally, these fluctuations frequently do not correlate well with measurable and known biomarkers, and available treatments are limited in their efficacy. This places patients and physicians in a challenging position.

Any single physician-patient encounter has the power to build either patient trust or patient mistrust, and this directly impacts the quality of care received and outcomes [29,30]. If the voices of patients with SLE are to be believed, many of them find themselves in a negative spiral whereby engaging with physician-provided care often fails to deliver the validation, support, and direction they need to live well with SLE. As a result, they may exit these encounters feeling confused, unheard, and unseen [30].

Insight on and progress with chronic disease management, in general, has been gained with the Chronic Disease Self-Management Program (CDSMP) [31]. The CDSMP is an intervention that was designed to support patients with chronic diseases when ongoing symptoms, such as fatigue and pain, negatively impact their ability to engage in the behaviors that they need to support their health (eg, exercise, healthy eating, effective communication strategies, and others) [31]. When tested on African American women with SLE, the CDSMP resulted in self-reported improvements in exercise, relaxation techniques, diet, and medication adherence [32]. Each of these areas touches multiple of the patient need domains described by Ra and colleagues [26] (Figure 1 shows an illustration of these domains). Unfortunately, the CDSMP requires participants to access a 6-week, live community workshop where these skills can be learned. This requirement creates a significant barrier to patient access [31]. However, awareness of the value of empowering patients in this way is impactful in and of itself. It helps validate the known limitations of our health care delivery system, the disproportionate impact of a single negative physician encounter for patients with SLE, and the opportunity for continuous access to support that is medically valid and endorsed.

## Digital Health Interventions and Social Listening

In the context of this report, a digital health intervention (DHI) was defined according to the following World Health Organization description: “a discrete functionality of digital technology that is applied to achieve health objectives” [33]. Over the last decade, interactive digital channels and social media platforms have emerged as meaningful sources of health and behavior data and have been leveraged in numerous studies covering many disease states. For instance, a 2017 study identified 137 published studies that were eligible for a systematic review of the use of health data from Twitter alone [34]. General social media platforms, such as Facebook, Weibo, and Reddit, and health specific platforms, such as PatientsLikeMe [20] and others, are demonstrating similar capabilities for identifying and articulating patient needs [35,36]. A recent analysis of a web-based survey, which was shared on multiple social media platforms, validated prior data showing that, broadly speaking, DHIs are most frequently leveraged by patients with SLE to understand the disease [37]. The most leveraged DHI for patients with SLE is the internet, primarily through the use of a smartphone or PC. Patients have reported using the internet for this purpose multiple times per day [26].

**Figure 2:**
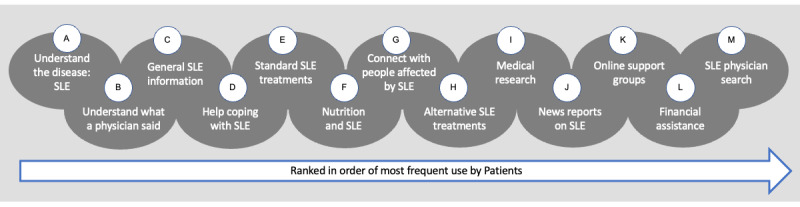
Patients’ opinions, which were gathered by Ra and colleagues [26], reveal their reasons for using DHIs and how they prioritize the use of DHIs. The reasons are alphabetized for use in Figure 3. DHI: digital health intervention; SLE: systemic lupus erythematosus.

Delving deeper to uncover additional specifics, through structured patient interviews and surveys, it was determined that the most common reasons for the use of a DHI among patients with SLE are as follows: to understand the disease better, to understand what their clinicians had told them, to access general information about SLE, to access help for coping with SLE, and to research the standard treatment options for SLE (Figure 2).

Additional reasons included the desire for social connection with other patients with SLE and online support groups [26]. Patients who are able to access social support demonstrate both improved health outcomes and improved health-related quality of life outcomes [38,39]. To derive this much needed support, patients use a variety of DHIs [26]. Patients have also reported wanting access to information in several different mediums and platforms. It is notable that in the study presented by Ra and colleagues [26], social support was not one of the top five reasons among patients with SLE for using DHIs. This indicates a gap in clinical encounters that is being addressed by the use of DHIs.

A recent analysis of health data that were generated by patients with SLE on Twitter concluded that social listening provides content that is clinically meaningful and relevant to the medical community. The study identified the following three areas of medical relevance, which were sourced from the authors’ content analysis: emerging patient needs, patient safety concerns, and adverse drug reactions [40,41]. The study also highlighted insights from diverse patients, whose voices are typically underrepresented [42,43]. There are additional studies underway for which the content analysis protocol has been published and final analyses are to soon follow [43]. A large study involving a social media content analysis of a web-based forum for a variety of chronic diseases, including SLE, indicated success in assessing patients’ desire to participate in studies and insights regarding their needs and priorities [44]. Additional studies have been published on the use of social listening on a publicly accessible, web-based forum—PatientsLikeMe [20]—to access and quantify both the needs of patients with SLE and their responses to various treatments [45-48]. All of these studies call out the value of these forums in helping to articulate patient need, with particular focus on patients’ understanding of SLE, interest in SLE research, treatment adherence (or lack thereof), and types of treatments used, among other insights.

Further validating this research is the growing recognition of the power of data-driven empathy. Acknowledged as a key pillar for transforming patient-centered care, DHIs and social listening have significant potential to inform data-driven empathy for both the individual and the population. Sloan and colleagues [30] conducted a thematic analysis to identify the following critical patient-centered themes through social listening and structured interviews: (1) the roles of the diagnostic process and key physician relationships in overall trust and well-being among patients with SLE; (2) the role of inadequate physician knowledge in patient distrust and changes in health care seeking behaviors, such as underreporting symptoms; and (3) the role of shared decision-making and its ability to build trust and mitigate insecurity.

**Figure 3:**
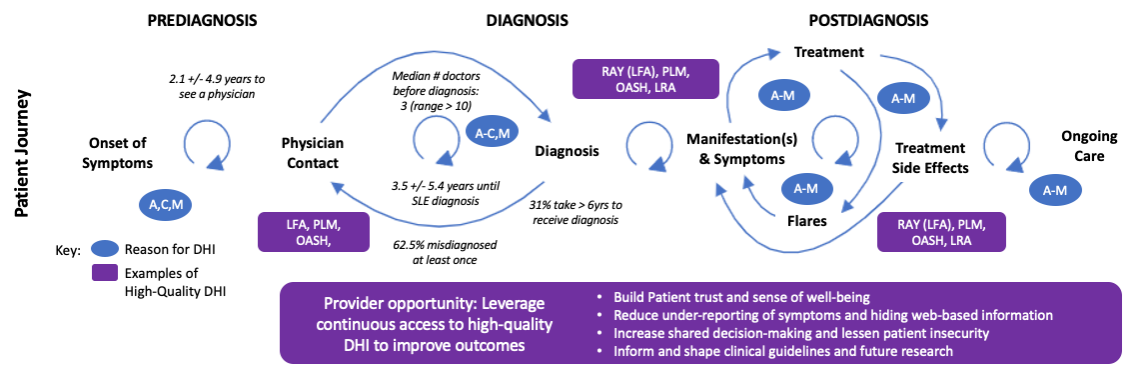
Patient digital voices reveal that patients with SLE frequently find themselves repeating loops within and between the prediagnosis, diagnosis, and postdiagnosis stages. Continuous access to clinician-supported DHIs [20-24,53] can result in the opportunity to validate the patient experience, better inform care, and improve outcomes. The reasons for using DHIs are sourced from Figure 2. DHI: digital health intervention; LFA: Lupus Foundation of America; LRA: Lupus Research Alliance; PLM: PatientsLikeMe; RAY: Research Accelerated by You; SLE: systemic lupus erythematosus; OASH: Office of Women’s Health.

## Opportunity

Our medical delivery system in the United States is inefficient and fragmented. Coupled with a multitude of health care access barriers, SLE represents a significant challenge for patients. To find answers to their questions and daily concerns, patients with SLE are depending on themselves to integrate the information they gather from their health care network, DHIs, and their community of caregivers. Typically, the integration of these additional sources of support occurs without supervision or medical oversight. Although the disconnect between clinical consensus guidelines, clinician recommendations, and how patients are addressing their needs is not apparent to clinicians, the gap is very real to patients with SLE (Figure 1 and Figure 2; Multimedia Appendix 1). Additionally, recent studies have shown that electronically captured PROMs are favored by patients and are perceived by patients as necessary for improving their care [27,49].

Clinicians, in general, have valid concerns about the quality of the information that their patients are receiving through DHIs. A recent review of these concerns is well summarized by O’Reilly-Jacob and colleagues [50]. The three main concerns were that DHI care is of low quality because it (1) is underdeveloped and thus ineffective or harmful, (2) is limited to supplementary care and is thus inefficient, and (3) is undesired because it does not align with clinician recommendations or true patient needs [41,50].

Despite clinician concerns, the general patient perspective is that the desire and opportunity for DHIs are tremendous. Conceptually, well-designed, monitored, and appropriately regulated DHIs could substitute, augment, or even deliver new care. Regardless of the quality of the care they are receiving, patients are reporting that they are actively searching for DHIs and are engaged in the use of DHIs to help them understand and manage their conditions on a daily basis [26]. O’Reilly-Jacob and colleagues [50] offer suggestions for how to address valid clinician concerns with low-quality, DHI-generated care. One suggestion is that medically recommended DHIs can be regulated by requiring evidence-based outcomes, appropriate risk labeling for approval, and tested protocols for implementing DHIs into the care pathway. These insights are valid; however, regulation will take years. Conversely, directing patients toward existing sources of valid content to address existing needs can be done in any encounter with a patient.

For clinicians to be comfortable with making DHI recommendations to their patients, they need confidence that the sources are reputable and that the DHI offers a safe and healthy environment for their patients [51]. Although not all existing DHIs are reputable and validated, there are several current sources (some are mentioned herein) that could be assessed and recommended by clinicians. It would benefit both clinicians and patients to openly align on which existing resources could be recommended. These conversations would create an opportunity to better understand individual patient preferences and needs for digital support. Guidelines could be instituted to assist clinicians in having these conversations with patients, matching individual patient needs with what current web-based resources can provide, and identifying what to watch out for (eg, determining when to not use web-based resources). In addition to benefiting from shared decision-making around how to use existing DHIs, patients would be contributing to improved future care by targeting high-quality, well-funded, and monitored resources.

Although the medical community continues to wrestle with the challenges they face regarding diagnosis and treatment for the multitude of SLE manifestations and flares, patients need immediate and ongoing access to resources that can help them navigate their individual, unpredictable lived experiences with SLE. Listening to the digital voices of patients with SLE has revealed a gap between the intermittent medical interventions that they receive and their lived continuum of needs. By leveraging existing digital resources, clinicians could recommend DHIs and proactively discuss with patients how they can support themselves with DHIs at every stage of the patient journey (Figure 3).

Clinicians could invite their patients to share which DHIs they are using and openly discuss which platforms are likely to most benefit patients and lupus research. The capturing, aggregation, and analysis of more complete individual patient experiences, as reported by patients themselves, would result in holistic patient-centered data that are rich with actionable insights.

Some work in this area—integrating the patient voice with current and future medical care while providing continuous access to high-quality DHIs—is underway with a quality of life digital toolkit [38,53] and a newer approach to patient registries. The Research Accelerated by You platform is a global registry of patients with SLE that is managed by the Lupus Foundation of America [22]. The intention of this DHI is to provide a forum where patients with SLE, their caregivers, and their families can enter information about their individual experiences regarding not only treatments but also overall patient and caregiver needs. The stated goal is to inform stakeholders about overall needs to drive research for improving the lived experiences of patients with SLE. The Lupus Foundation of America [24] is a well-funded organization of good repute. It includes LupusConnect, which is a medically moderated patient support group and web-based lupus community [21]. Lupus experts could consider evaluating 1 or 2 respected DHIs that their patients could be directed toward, depending on their patients’ current needs (eg, PatientsLikeMe [20], Lupus Research Alliance [52], the *Lupus* section of the Office of Women’s Health [23], and others). These will not be static over time. Clinicians will need to identify and monitor 1 or 2 resources that they feel comfortable recommending and adjust this recommendation as needed on an ongoing basis.

The viewpoint presented herein is that patients with lupus are sourcing their medical information from digital media for even the most basic of reasons to understand the disease. This indicates an immediate opportunity. Mutual openness; shared language; and shared decision-making for patients’ use of DHIs, wherein patients work with their clinicians to address the mutual gaps in their knowledge, have the potential to result in improved and more patient-centered care. Initiating conversations regarding the details of individual patients’ use of DHIs will result in the integration of patients’ comprehensive lived experiences into encounters with health care professionals; patients will share more of their experiences and symptoms that clinicians cannot yet measure and cannot yet treat. A mutual acceptance of the unknown is much more likely to build rather than compromise patient trust and well-being.

This viewpoint paper is targeted to patients with lupus. However, the perspectives and opportunities presented herein are not limited to patients with lupus. Patients with chronic fatigue syndrome, Lyme disease, Crohn disease, and other “invisible” diseases follow a similar pattern of sourcing the support that they need from DHIs. Ultimately, listening to patients requires humility and openness to information that is potentially not available in existing medical guidelines but can be leveraged to improve and shape existing and future care.

## Appendix

Multimedia Appendix 1 Word cloud content sourced from 341,372 members (68,581 systemic lupus erythematosus symptoms recorded) on PatientsLikeMe. The word cloud was constructed with WordItOut (Enideo) [28].

## Abbreviations

CDSMP: Chronic Disease Self-Management Program
DHI: digital health intervention
PROM: patient-reported outcome measure
SLE: systemic lupus erythematosus
