# Supplementary figures and images for: Listening to Patients With Lupus: Why Not Proactively Integrate the Internet as a Resource to Drive Improved Care?
Source: J Med Internet Res. 2023 Mar 29;25:e44660. doi: 10.2196/44660 (PMC10131912; doi:10.2196/44660)

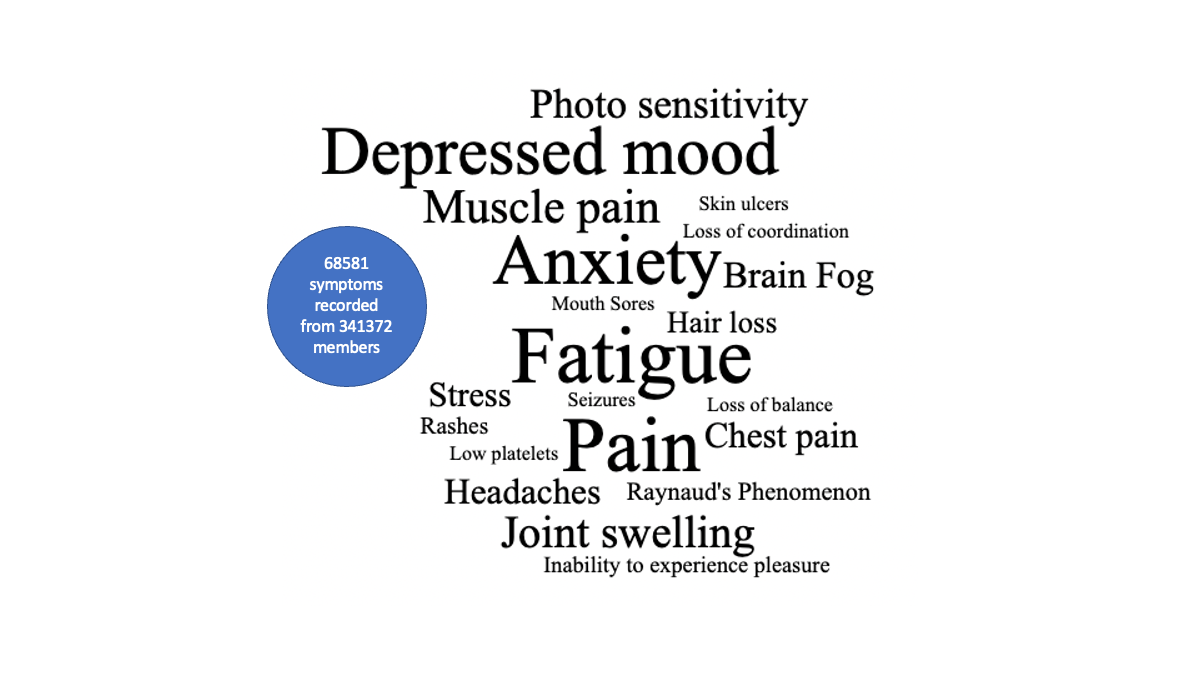

Supplement: Multimedia Appendix 1 [file jmir_v25i1e44660_app1.png]
